# Supplementary material for: Evidence supported by Mendelian randomization: impact on inflammatory factors in knee osteoarthritis
Source: Front Med (Lausanne). 2024 May 28;11:1382836. doi: 10.3389/fmed.2024.1382836 (PMC11165061; doi:10.3389/fmed.2024.1382836)
Supplement: Supplementary file 1 [file Data_Sheet_1.ZIP › data/GCST90274831_5e6/831.leaveoneout.pdf]

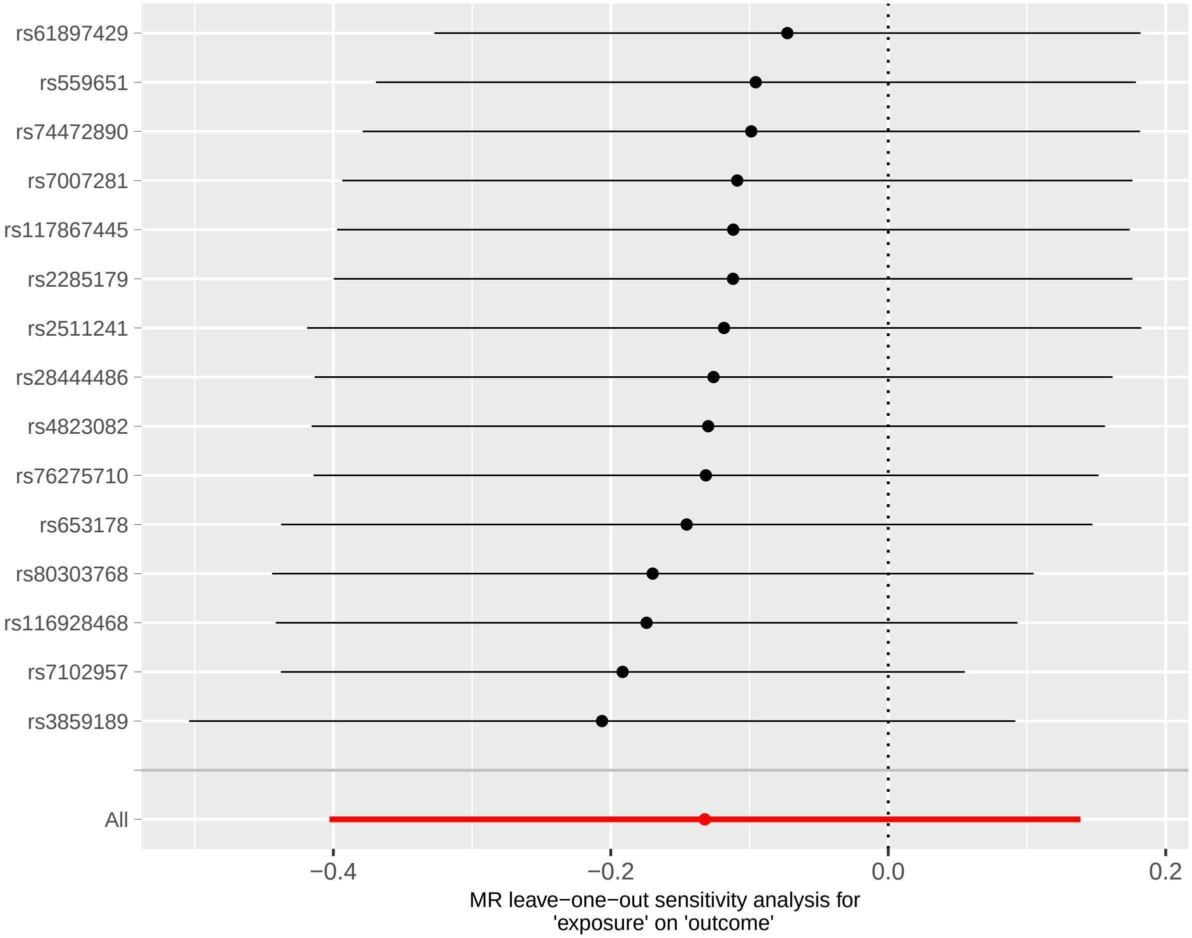
rs61897429
rs559651
rs74472890
rs7007281
rs117867445
rs2285179
rs2511241
rs28444486
rs4823082
rs76275710
rs653178
rs80303768
rs116928468
rs7102957
rs3859189
All
−0.4
−0.2
0.0
0.2
MR leave−one−out sensitivity analysis for
'exposure' on 'outcome'
